# Expectations About Future Economic Prospects and Satisfaction with Democracy: Evidence from European Countries during the COVID-19 Crisis

**DOI:** 10.1007/s11205-021-02783-8

**Published:** 2021-08-26

**Authors:** Elina De Simone, Lorenzo Cicatiello, Giuseppe Lucio Gaeta, Mauro Pinto

**Affiliations:** 1 Department of Economics, Roma Tre University, Via Silvio D’Amico 77, 00145 Rome, Italy; 2 Department of Mathematics, Computer Science and Economics, University of Basilicata, Via dell’Ateneo Lucano 10, 85100 Potenza, Italy; 3 Department of Human and Social Sciences, University of Naples L’Orientale, Largo San Giovanni Maggiore 30, 80134 Naples, Italy; 4 Department of Political Sciences “Jean Monnet”, University of Campania Luigi Vanvitelli, Viale Ellittico 31, 81100 Caserta, Italy

**Keywords:** Satisfaction with democracy, Subjective expectations, COVID-19 disease, Government support measures

## Abstract

Recent studies highlight that economic expectations are a crucial determinant of citizens’ satisfaction with democracy (SWD). This article relies on a cross-sectional analysis of European survey data collected in the aftermath of COVID-19 disease to investigate the relationship between citizens’ expectations about future economic prospects and their SWD. Our findings support the idea that citizens’ expectations about future economic prospects are correlated with SWD. Furthermore, they reveal that perceived conditions of material wellbeing moderate this relationship.

## Introduction

It is widely acknowledged that the spread of the COVID-19 virus put democracies under pressure (e.g., Bol et al., 2020; Flinders, 2020). Many European agencies and think tanks have launched surveys to investigate the possible implications of the pandemic across Europe (e.g., Eurofound; European Council on Foreign Relations—ECFR),^1^ exploring how the outbreak has shaped European citizens’ attitudes toward the governing authorities. This topic is considered highly relevant because “the coronavirus is already reshaping the debate about regime legitimacy and state capacity” (Flinders, 2020 p. 17).

In this turbulent context, an inquiry into citizens’ attitudes towards democracy is deemed essential, especially in Europe, where dissatisfaction with democracy seems to rise (Kriesi, 2020). In detail, the choice of exploring people’s satisfaction with democracy (SWD) would make much sense because this latter “is probably the most important element for the success of a democratic system” (Chang, 2018, p. 999).

In line with this perspective, our paper uses data collected during the pandemic to examine the correlates of citizens’ SWD. More specifically, by using cross-sectional microdata collected by Eurofound (2020) in its survey of European citizens’ living and working conditions in the wake of COVID-19, our investigation aims to assess whether individuals’ prospective expectations about their economic situation are correlated with their SWD.

The motivation of such a focus on the link between expectations about future economic prospects and SWD is two-fold. On the one hand, while there is broad consensus about the idea that economic conditions matter for democracy (Dahl, 1989; Lipset, 1994), specific evidence on the link between individual expectations about the economy and SWD remains limited, even if attention for this topic is increasing (Loveless & Binelli, 2020; Nadeau et al., 2019). On the other hand, a growing body of research reveals that the pandemic had massive effects on economic sentiment and expectations (e.g., Fetzer et al., 2020), which suggests checking whether these expectations, in turn, exert any influence on SWD.

As far as we know, this paper is one of the first attempts to explore the importance of expectations about future economic prospects on SWD in the context of the COVID-19 outbreak. By testing the link between these two variables, our analysis controls for government support measures directed towards citizens and for trust in institutions, which allows us to provide additional findings that contribute to the discussion on the role of policy decisions and evaluations of institutions in explaining political attitudes during the COVID-19 pandemic (Bol et al., 2020; Naumann et al., 2020; Schraff, 2020).

The paper is organized as follows. Section two briefly reviews the most recent literature relevant to our study. Section three presents the data and methodology applied to analyze them. Section four presents our findings. Finally, Section five provides the results’ discussion and conclusion.

## Background and hypotheses

Scholars claim that objective economic conditions and their subjective perception have a remarkable impact on citizens’ SWD (Quaranta & Martini, 2016 and 2017; Christmann & Torcal, 2017; Daoust & Nadeau, 2020).

The most recent literature adds that citizens’ prospective economic expectations are also crucial SWD drivers. Nadeau et al. (2019) develop a forward-looking theory that is primarily rooted in the behavioral economics models of expectations’ formation and the “prospect of upward mobility” (POUM) hypothesis (Benabou & Oak, 2001). According to them, people who expect to experience better economic conditions are characterized by a sort of “wishful thinking” and have an “optimism bias” or “positive illusions,” which make them more prone to a positive evaluation of institutional performance. This thesis is supported by an empirical exercise–based on the Comparative Study of Electoral Systems (CSES) survey data–showing that a relationship between citizens’ expectations about future economic prospects and their evaluation of democracy performance exists and is conditioned by individual wealth.

In the same line of research, Loveless and Binelli (2020) analyze jobless skilled young Italian individuals and find that expectations about future economic prospects—i.e., about job stability, job security, and earnings—affect their satisfaction with the way democracy works in the country.

This strand of research has gained significant relevance during the COVID-19 crisis since the pandemic had a remarkable impact on people’s sense of economic vulnerability and produced effects on economic perceptions and expectations (Fetzer et al., 2020; Mann et al., 2020). In such a context, an inspection of the strength of the link between expectations about future economic prospects and SWD seems particularly appropriate. Surprisingly, the literature that explored the drivers of citizens’ political support during the pandemic has not considered the role of prospective economic expectations in influencing the opinions about institutional performance (e.g., Bol et al., 2020; Schraff, 2020).

Drawing on the SWD research recalled above, we formulate the following hypothesis to be tested in our empirical analysis:

- *H1* Optimistic (pessimistic) expectations about future economic prospects exert a positive (negative) impact on citizens’ opinions regarding the performance of a democratic system (the prospective economic evaluation hypothesis).

Scholars have added to this picture by suggesting that material wellbeing moderates the effect of economic expectations on SWD. “The intuition is straightforward: material gain should matter more to the poor because it leads to a larger (relative) improvement in living standards for them than for the already rich” (Nadeau et al., 2019, p. 1081). Conversely, bad economic prospects should decrease SWD more among the poor. In other words, we expect the effect of prospective economic consequences on SWD not to be homogeneous across individuals but to vary according to subjective economic conditions. We, therefore, formulate the following additional hypothesis:

- *H2* The effect of expectations about future economic prospects on SWD is greater among poorer people.

To study the ceteris paribus link between economic prospects and SWD, our analysis must consider the role of trust in governing authorities and the effect of support measures on SWD. Citizens’ support of the performance of a democratic regime is highly sensitive to different institutional contexts (Linde & Ekman, 2003). The inclusion of trust as a possible correlate of SWD can, thus, be justified in the light of the input-oriented procedural model of regime support, where “support is based on trust in democratic institutions as well as participation in the democratic process” (Hoboldt, 2012, p. 93). Earlier studies already document a positive correlation between trust in institutions and SWD (e.g., Hoboldt, 2012; Christmann & Torcal, 2017); trust appears as a significant predictor of SWD in times of crisis (Vlachová, 2019). A recent and still growing COVID-19 related literature confirms the decisive role of trust in governing institutions in conditioning citizen evaluations of government activity during the pandemic (Devine et al., 2020; Altiparmakis et al., 2021). The motivation lies in the ‘rally around the flag’ effect, which, playing as a “mechanism of retrospective performance evaluation” of the policies adopted in response to the coronavirus crisis, is found to affect citizens’ democratic attitudes (Bol et al., 2020, p.2). Increased perceptions of threats “shape the dynamics of public opinion” (Kritzinger et al., 2021, p. 1223). On the one hand, increased trust in government is a consequence of the empowerment of leaders as a bulwark against the virus-related threats when citizens ask for more protection (Bækgaard et al., 2020). On the other hand, it also represents people’s solutions to offset the uncertainty and distress related to the pandemic turmoil (Kritzinger et al., 2021).

According to the output-oriented performance model of regime support (Hoboldt, 2012), SWD may depend on how citizens evaluate government performance. In the context of coronavirus disease, as governments were asked to adopt response measures to tackle the crisis's adverse effects, performance evaluation is, thus, related to citizens’ assessment of government responses to COVID-19 (Altiparmakis et al., 2021). In detail, the adoption of support measures, shaping the way people think about the pandemic, is supposed to affect people’s risk perception and capacity to bear the effects of the disease (Naumann et al., 2020, p. 2). A citizen who is a recipient of a support measure is more likely to judge how democracy works in his country positively. Furthermore, the better people rate the government responses, the higher their reported satisfaction with democracy is. “Policy evaluations are part and parcel of democratic representation”, they contribute to a more compliant attitude over time by citizens, which is the core ingredient of success (Altiparmakis et al., 2021, p. 1162). This, in turn, conditions the regime’s legitimacy, which “is largely determined by its effectiveness to deliver goods to the public” (Linde & Ekman, 2003, p. 400). As citizens asked governments to provide an adequate set of public goods to counteract the consequences of the pandemic, once such goods were delivered, the more effective a government was at delivering such goods, the better the people valued the performance of democracy.

## Data and methodology

Our analysis relies on the micro-data provided by round two of the Eurofound’s “Living, Working and COVID-19” survey (2020), carried out in the EU27 countries between June and July 2020.

Since respondents were recruited via uncontrolled convenience sampling (2020),^2^ all the individual responses were re-weighted to represent each respondent’s country’s demographic. Following Eurofund’s instructions, we used the weight provided by the dataset (*w_gross_trim*). According to the dataset codebook, the provided weights “can be used for both within-country and cross-country analysis” (Sandor, 2020, p.1).

The survey includes one question asking respondents to indicate their SWD in their country, our dependent variable. Replies are coded by one ordered variable whose values range from 0 (*very dissatisfied*) to 10 (*very satisfied*). This original variable has been re-coded into three categories (low, medium, high satisfaction) to ease the interpretation of our results.^3^

The observed *i*th respondent SWD is modeled as a function of two main variables: *economic_prospects* and *spending_ability*.

Expectations about future *economic_prospects* is a categorical variable that measures whether the respondent feels that his/her household financial situation in three months will be *better*, the *same,* or *worse* than it is at present. We expect that better economic prospects are connected with higher evaluations of democracy performance.

*Spending ability* is one ordinal variable measuring the respondents’ household’s ability to make ends meet in regular pre-COVID times. This variable should convey information on the respondent’s material wellbeing, which is supposed to condition the link between individual economic prospects and SWD.^4^

The analysis considers four groups of control variables. The first group of covariates includes socio-demographic controls such as respondent’s age (*youth*), gender (*male*), household composition (*couple*, *parent_granparents*, *child_in_hh*), education (*education*), employment status (*emp_stat,* distinguishing among *employee, self-employed, unemployed, retired, other*) and self-perceived health (*bad_health*).

The second group of variables observes respondents’ financial and living conditions by measuring their household’s financial situation during the last three months (*worse_householdeconomy)* and whether they feel that they would leave their current accommodation due to economic problems over the following six months (*hou_insec*).

The third group includes variables controlling for individual trust in the government (*trust_gov*) and national health system (*trust_health*), which we argue are essential to complement trust in institutions in the specific context of the COVID-19 pandemic.

The fourth group includes the categorical variables that observe respondents’ perceptions about the support measures applied by their national government in the specific context of the COVID-19 pandemic. More precisely, *measures_fair* indicates (in a range from 1 to 5) the perceived fairness of support measures, and *measures_destinatary* indicates in a range from 1 to 5 how much the respondent agrees with the idea that support measures reach those who need them most. These variables concern the assessment of the current government and its intervention. Moreover, this group also includes one set of dummies indicating whether the respondent has received any public financial support since the outbreak of the COVID-19 pandemic (*support_unemp; support_wage; support_sickleave; support_payments; support_other*). Finally, the analysis considers country dummies controlling for unobserved cross-country heterogeneity.

All the variables used in the analysis are presented in Table 1, where basic descriptive statistics are also reported. The original survey data include 24,123 observations. Unfortunately, some observations report missing values for some of the variables that we are interested in. As a consequence of listwise deletion, the sample under scrutiny in our analysis has fewer observations (15,073) in some specifications. All the analyses use the observations’ weights provided by the “Living, Working and COVID-19” survey to ensure that the data represent the countries’ demographic profiles.

**Table 1:** Label, description and summary statistics of the variables used in the study

Variable	Modality	Description	N	Mean	Std. Dev	Min	Max
SWD	Low	Dummy = 1 if satisfaction with democracy is low	20,620	0.322	0.467	0	1
	Medium	Dummy = 1 if satisfaction with democracy is medium	20,620	0.438	0.496	0	1
	High	Dummy = 1 if satisfaction with democracy is high	20,620	0.241	0.427	0	1
Expectations about future Economic_prospects	Better ( +)	Dummy = 1 if expected household financial situation in 3 months is better than now	20,620	0.116	0.320	0	1
	The same	Dummy = 1 if expected household financial situation in 3 months is the same then now	20,620	0.634	0.482	0	1
	Worse	Dummy = 1 if expected household financial situation in 3 months is worse than now	20,620	0.250	0.433	0	1
Emp_stat	Employee	Dummy = 1 if employment status is employee	20,620	0.431	0.495	0	1
	Self-employed	Dummy = 1 if employment status is self-employed	20,620	0.071	0.256	0	1
	Unemployed ( +)	Dummy = 1 if employment status is unemployed	20,620	0.092	0.289	0	1
	Retired	Dummy = 1 if employment status is retired	20,620	0.274	0.446	0	1
	Other	Dummy = 1 if employment status is other	20,620	0.132	0.338	0	1
Bad_health		Dummy = 1 if the interviewed health status is bad	20,620	0.076	0.265	0	1
Education	Primary ( +)	Dummy = 1 if interviewed education level is primary	20,620	0.051	0.220	0	1
	Secondary	Dummy = 1 if interviewed education level is secondary	20,620	0.658	0.474	0	1
	TERTIARY	Dummy = 1 if interviewed education level is tertiary	20,620	0.291	0.454	0	1
Youth		Dummy = 1 if interviewed is aged 18–29 years	20,620	0.154	0.361	0	1
Male		Dummy = 1 if gender is male	20,620	0.479	0.500	0	1
Couple		Dummy = 1 if interviewed household includes a partner/spouse	20,620	0.596	0.491	0	1
Parent_granparents		Dummy = 1 if interviewed household includes parents or grandparents	20,620	0.145	0.352	0	1
Child_in_hh		Dummy = 1 if interviewed household includes children aged 0–11 years	20,620	0.238	0.426	0	1
Hou_insec		Dummy = 1 if it is likely that interviewed will need to leave accommodation in 6 months	19,370	0.046	0.209	0	1
Worse_householdeconomy		Dummy = 1 if household financial situation 3 months ago was better than now	19,370	0.317	0.465	0	1
Spending_ability	With great difficulty ( +)	Dummy = 1 if household is normally able to make ends meet with great difficulty	19,370	0.089	0.285	0	1
	With difficulty	Dummy = 1 if household is normally able to make ends meet with difficulty	19,370	0.110	0.313	0	1
	With some difficulty	Dummy = 1 if household is normally able to make ends meet with some difficulty	19,370	0.208	0.406	0	1
	Fairly easily	Dummy = 1 if household is normally able to make ends meet fairly easily	19,370	0.261	0.439	0	1
	Easily	Dummy = 1 if household is normally able to make ends meet easily	19,370	0.201	0.401	0	1
	Very easily	Dummy = 1 if household is normally able to make ends meet very easily	19,370	0.130	0.336	0	1
Support_unemp		Dummy = 1 if interviewed has received any unemployment benefit since the outbreak of pandemic	19,370	0.050	0.218	0	1
Support_wage		Dummy = 1 if interviewed has received any wage support since the outbreak of pandemic	19,370	0.057	0.231	0	1
Support_sickleave		Dummy = 1 if interviewed has received any sick/care leave support since the outbreak of pandemic	19,370	0.035	0.185	0	1
Support_other		Dummy = 1 if interviewed has received other forms of support since the outbreak of pandemic	19,370	0.042	0.200	0	1
Support_payments		Dummy = 1 if interviewed has received any kind of support related to tax, bill, mortgage, loan or debt since the pandemic	19,370	0.050	0.218	0	1
Measures_fair	Strongly agree ( +)	Dummy = 1 if interviewed strongly agree with fairness of support measures introduced during the pandemic	15,073	0.031	0.174	0	1
	Agree	Dummy = 1 if interviewed agree with fairness of support measures introduced during the pandemic	15,073	0.191	0.393	0	1
	Neither agree nor disagree	Dummy = 1 if interviewed neither agree nor disagree with the fairness of support measures introduced during the pandemic	15,073	0.280	0.449	0	1
	Disagree	Dummy = 1 if interviewed disagree with the fairness of support measures introduced during the pandemic	15,073	0.255	0.436	0	1
	Strongly disagree	Dummy = 1 if interviewed strongly disagree with the fairness of support measures introduced during the pandemic	15,073	0.243	0.429	0	1
Measures_destinatary	Strongly agree ( +)	Dummy = 1 if interviewed strongly agree with the rightness of destination of support measures introduced during the pandemic	15,073	0.027	0.163	0	1
	Agree	Dummy = 1 if interviewed agree with the rightness of destination of support measures introduced during the pandemic	15,073	0.176	0.381	0	1
	Neither agree nor disagree	Dummy = 1 if interviewed neither agree nor disagree with the rightness of destination of support measures in the pandemic	15,073	0.283	0.450	0	1
	Disagree	Dummy = 1 if interviewed disagree with the rightness of destination of support measures introduced during the pandemic	15,073	0.265	0.442	0	1
	Strongly disagree	Dummy = 1 if interviewed strongly disagree with the rightness of destination of support measures during the pandemic	15,073	0.249	0.432	0	1
Trust_health		Dummy = 1 if trust in healthcare system is high	15,073	0.686	0.464	0	1
Trust_gov		Dummy = 1 if trust in country’s government is high	15,073	0.420	0.494	0	1

Given the ordered nature of our SWD variable, our estimates are carried out through an ordered logit model. The cross-sectional nature of our empirical exercise prevents the identification of any causal link among the variables. Such cross-sectional studies have to face the hurdles of omitted variables, endogeneity, and the potential issue of reverse causality. To avoid the omitted variable bias, we choose an extensive set of control variables related to the outcome (SWD) and our independent variables of interest (Daoust & Nadeau, 2020) and include them progressively in our specifications. In this way, if the sign and significance of our primary independent variable are consistent among the different specifications, we may reasonably discard that our results depend on omitting relevant variables.

Previous studies addressed the concern of endogeneity using an instrumental variables approach. Economic perceptions have been instrumented with a number of variables, such as individual socio-economic status (e.g. race, gender, education, income, interest in politics, job status and union membership in Lewis-Beck et al., 2008) or macroeconomic figures (e.g. inflation, GDP growth, unemployment rate in Nadeau & Lewis-Beck, 2001; and Bellucci & Lewis-Beck, 2011). It would be hard to sustain that the variables of the first group are a valid instrument in our study, as they are directly correlated to SWD (see the results for gender, education or employment status in the next section). Macroeconomic variables instead, besides they could be also directly correlated to SWD (e.g. worsened economic performance may lead to dissatisfaction towards democracy), cannot be included in our specification due to the lack of heterogeneity within country. In short, previously used instruments fail the exclusion restriction or are not suitable for the present study. Therefore, in the absence of a valid instrument, the estimates cannot be interpreted as casual but just as robust, *ceteris paribus* correlations.

First of all, generally pessimistic people will tend to report poor economic prospects and low SWD. The inclusion of employment status and a variable on declared spending ability of the respondents, mirroring the objective economic status of the respondents (Lewis-Beck, 2006), should help downsize the issue of omitted variable. Furthermore, in countries where democracy is not working well, that may cause people to believe their economic prospects are worse. In times of Covid-19, a possible failure of democracy is considered to be highly related to governments’ response measures to the crisis and trust between governors and the governed (e.g., Bol et al., 2020; Devine et al., 2020, Altiparmakis et al., 2021). The inclusion of trust in government variables and respondents’ reception and assessment of governmental supporting measures were aimed at smoothing such concerns. Finally, where the pandemic was especially bad, governments might have provided more support, but democracy may have eroded more. Heterogeneity in epidemiological cases of Covid-19 should be captured by including country-fixed effects in the model. Furthermore, the inclusion of a variable catching the respondent's health status should also help control the incidence of the pandemic at the individual level.

## Results

### Main findings

Table 2 shows the estimates obtained through our regression analyses. Five specifications are reported. In model (1), we test H1 by including individuals' expectations about future economic prospects as regressors alongside the entire set of socio-demographic controls. To test the robustness of the findings obtained through this model, we include additional covariates in the (2)–(4) specifications. In model (2), we add the spending ability variable alongside covariates controlling economic prospects and socio-demographics. Model (3) adds the variables observing the respondents’ perception of government policy measures, while model (4) includes the trust-related covariates. Finally, to test H2, in model (5), the interaction between respondents’ expectations about future economic prospects and spending ability is explored.

**Table 2:** Ordered Logit estimates

	(1)	(2)	(3)	(4)	(5)
*Expectations about future economic_prospects*					
The same	− 0.083*	− 0.161***	− 0.086	0.027	− 0.127
	[0.044]	[0.047]	[0.055]	[0.057]	[0.236]
Worse	− 0.968***	− 0.785***	− 0.553***	− 0.369***	− 0.791***
	[0.050]	[0.053]	[0.062]	[0.065]	[0.233]
*Employment_status*					
Employee	0.557***	0.053	− 0.085	0.052	0.068
	[0.052]	[0.062]	[0.069]	[0.074]	[0.074]
Self-employed	0.251***	− 0.178**	− 0.221**	− 0.077	− 0.073
	[0.071]	[0.079]	[0.088]	[0.093]	[0.093]
Retired	0.721***	0.258***	− 0.013	0.098	0.113
	[0.056]	[0.066]	[0.076]	[0.080]	[0.081]
Other	0.564***	0.103	− 0.155*	− 0.143*	− 0.120
	[0.061]	[0.069]	[0.080]	[0.085]	[0.086]
Bad_health	− 0.663***	− 0.492***	− 0.275***	− 0.115	− 0.094
	[0.055]	[0.059]	[0.068]	[0.072]	[0.072]
Secondary education	0.378***	0.258***	0.417***	0.385***	0.387***
	[0.066]	[0.072]	[0.081]	[0.083]	[0.084]
Tertiary education	0.675***	0.430***	0.556***	0.358***	0.363***
	[0.069]	[0.075]	[0.086]	[0.088]	[0.089]
Youth	0.293***	0.290***	0.304***	0.319***	0.305***
	[0.046]	[0.048]	[0.058]	[0.061]	[0.062]
Male	− 0.153***	− 0.221***	− 0.189***	− 0.192***	− 0.200***
	[0.027]	[0.029]	[0.034]	[0.036]	[0.036]
Couple	0.150***	0.058*	0.041	0.043	0.044
	[0.031]	[0.032]	[0.039]	[0.041]	[0.041]
Parent_granparents	0.228***	0.073	0.139**	0.092	0.102*
	[0.045]	[0.048]	[0.056]	[0.059]	[0.059]
Child_in_hh	− 0.037	0.041	0.126***	0.182***	0.183***
	[0.034]	[0.036]	[0.041]	[0.044]	[0.044]
Hou_insec		− 0.026	− 0.044	− 0.083	− 0.082
		[0.078]	[0.086]	[0.091]	[0.092]
Worsed_householdeconomy		− 0.176***	− 0.098**	− 0.058	− 0.063
		[0.036]	[0.042]	[0.044]	[0.045]
*Spending_ability*					
Difficulty		0.396***	0.258***	0.366***	0.213
		[0.069]	[0.079]	[0.082]	[0.256]
Some difficulty		0.544***	0.231***	0.364***	0.079
		[0.065]	[0.075]	[0.078]	[0.249]
Fairly easily		1.006***	0.450***	0.496***	0.307
		[0.067]	[0.077]	[0.080]	[0.240]
Easily		1.117***	0.528***	0.452***	0.112
		[0.071]	[0.082]	[0.086]	[0.239]
Very easily		1.236***	0.530***	0.497***	0.006
		[0.077]	[0.091]	[0.095]	[0.251]
Support_unemp		− 0.179**	− 0.401***	− 0.608***	− 0.608***
		[0.070]	[0.077]	[0.082]	[0.082]
Support_wage		0.234***	0.057	0.023	0.008
		[0.063]	[0.070]	[0.074]	[0.074]
Support_sickleave		0.473***	0.502***	0.668***	0.664***
		[0.076]	[0.088]	[0.094]	[0.094]
Support_other		0.421***	0.504***	0.275***	0.296***
		[0.072]	[0.081]	[0.086]	[0.087]
Support_payments		0.370***	0.043	0.028	0.050
The same#difficulty		[0.069]	[0.077]	[0.081]	[0.081]
					− 0.189
The same#some difficulty					[0.285]
					0.068
The same#fairly easily					[0.271]
					0.063
The same#easily					[0.261]
					0.296
The same#very easily					[0.260]
					0.459*
Worse#difficulty					[0.273]
					0.505*
Worse#some difficulty					[0.283]
					0.616**
Worse#fairly easily					[0.273]
					0.281
Worse#easily					[0.267]
					0.142
Worse#very easily					[0.275]
					0.038
					[0.347]
_cons	− 0.852***	− 0.680***	− 2.867***	− 0.671***	− 0.953***
	[0.128]	[0.145]	[0.205]	[0.219]	[0.300]
_cons	1.386***	1.643***	− 0.199	2.902***	2.627***
	[0.129]	[0.146]	[0.203]	[0.222]	[0.302]
Country dummies	yes	yes	yes	yes	yes
R2 Adj	0.102	0.103	0.200	0.323	0.325
N	20,620	19,370	15,084	15,073	15,073

Coefficients and standard errors (in parentheses). The dependent variable is an ordered variable measuring satisfaction with democracy (SWD) and ranging from 1 (low) to 3 (high)

***p < 0.01. **p < 0.05. *p < 0.1

Looking at the estimates, the first evident result is that we find a statistically significant (*p* < 0.001) negative correlation between pessimistic economic expectations about economic prospects and SWD. In other words, having the feeling that in three months the household financial situation will be worse than today (instead than better than today) translates into lower SWD. This finding is highly consistent across the models and strongly supports H1. In specifications (1 and 2), we also find that those who feel that their future economic condition will be unaltered compared with today report a lower SWD than those who are optimistic. Also this finding is consistent with H1, but is not confirmed by the other specifications.

**Fig. 1:**
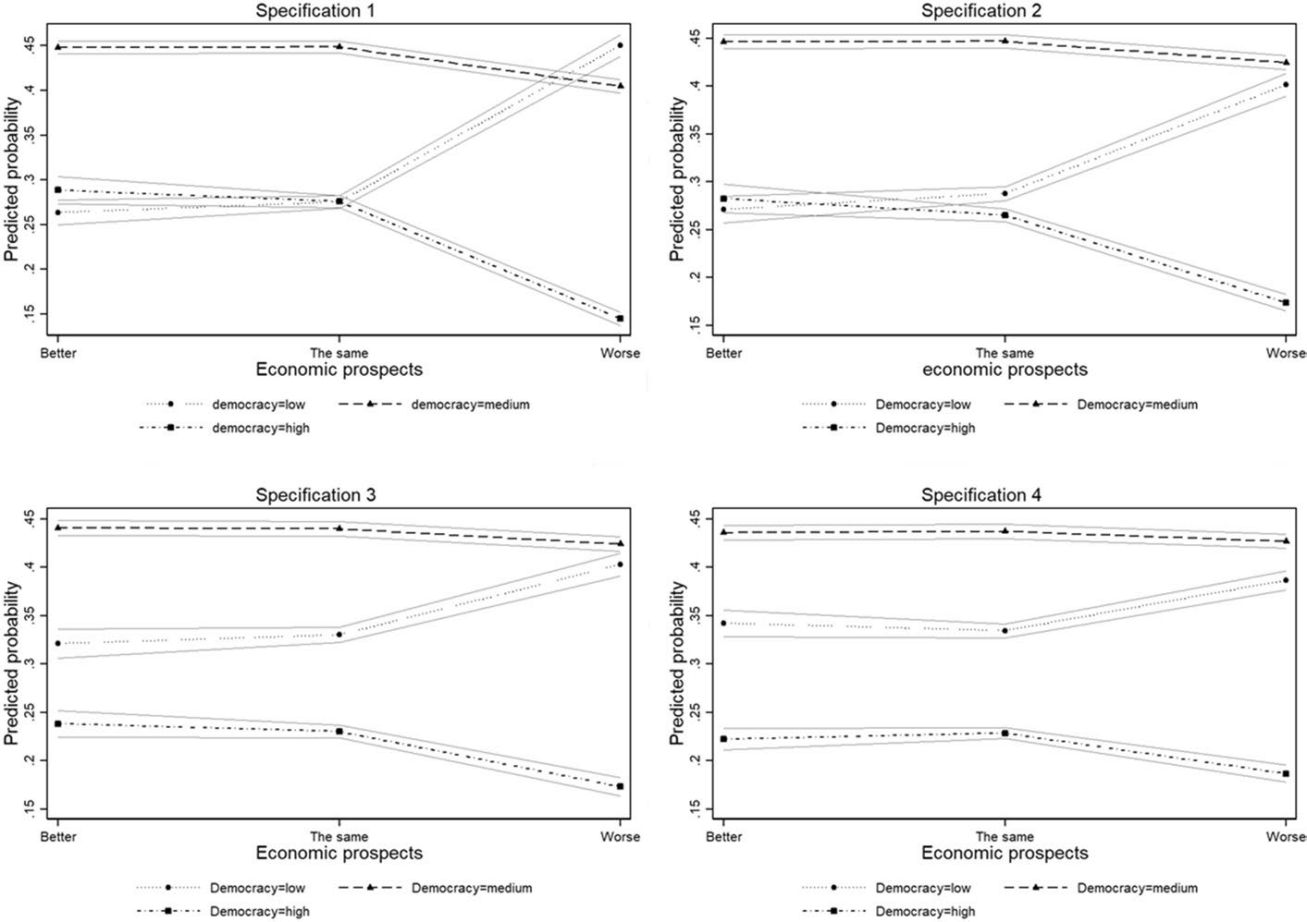
Predicted probability of SWD (low-medium–high) at different levels of expectations about future economic prospects (First, second, third, and fourth specification)

In Fig. 1 we plot the predicted probabilities of SWD for the different levels of economic prospects (better, the same, worse) as resulting from the estimation of model (4). These figures show that generally a medium SWD is the most likely outcome. As the economic prospects worsen, the predicted probability of low SWD increases, becoming the highest (specification 1) or about equal to medium SWD (specification 2, 3, and 4). Instead, the predicted probability of a high SWD decreases as the economic prospects worsen, reaching a minimum of about 0.15 in all four specifications. Therefore, all else equal, a shift from better to worse economic prospects decreases the likelihood of expressing high SWD and a corresponding increase of expressing low SWD.

Two additional findings are worth noting. First, the effect of expectations about future economic prospects on the probability of showing an intermediate SWD (*SWD* = *medium*) seems less remarkable. Second, while the magnitude of the *worse* impact on SWD is sizeable in our more parsimonious models, our more complete specification, especially when trust in institutions is considered an additional covariate (model 4), suggests that it is less remarkable.

On the whole, these estimates support the idea that during the COVID-19 crisis, pessimistic feelings about economic prospects lead to dissatisfaction with democracy. This is particularly true when we assess moving from good (*better*) to bad (*worse*) prospects.

### Other findings

The results obtained for the control variables provide insights that seem to be potentially valuable. Some aspects of family composition (*youth*, *parent_granparents*) and education show a positive correlation. At first, the magnitude of such an effect appears higher for *tertiary education*, while it significantly lows as an effect of the progressive inclusion of covariates (model (3)(4)). A positive correlation is also found for the *couple* and *child_in_hh* variables. However, while the latter is statistically significant only in the specification (3), (4) and (5), *couple* loses statistical significance due to covariates' inclusion. The economic controls suggest that the worse the current economic situation, the lower individual SWD. A recent worsening of a household’s financial condition (*worse_householdeconomy*) has a negative and statistically significant correlation with the dependent variable. At the same time, an increasingly positive impact on SWD is correlated with the more effortless *spending ability.* It is worth noting that *worse_householdeconomy* loses statistical significance when *trust* is included in the analysis, confirming the role played by trust in counteracting feelings of economic threats during the pandemic (Kritzinger et al., 2021). Receiving sick leave support or other forms of support has a positive correlation with satisfaction with democracy. *Wage support* and *payment support* have a positive effect, but statistical significance is observed only in specification (1).

Meanwhile, the reception of support measures for unemployment appears to be negative and statistically significant. *Measures_fair* and *measures_destinatary* report p-values below commonly-used thresholds. A final point concerns the effects of trust variables, which, consistently with recent literature (Bol et al., 2020), register a high impact on SWD in times of COVID-19 too. In specification (4), *trust_health* and *trust_gov* show a highly statistically significant positive *ceteris paribus* correlation with the dependent variable (*p* < 0.001). As already been highlighted, when such controls are included in the estimations, the size of the effects of pessimistic expectations about economic prospects is meaningfully reduced compared to previous specifications. This result is in line with the rallying around the flag effect in times of Covid-19 (Bækgaard et al., 2020).

### Expectations about future economic prospects and current economic conditions

**Fig. 2:**
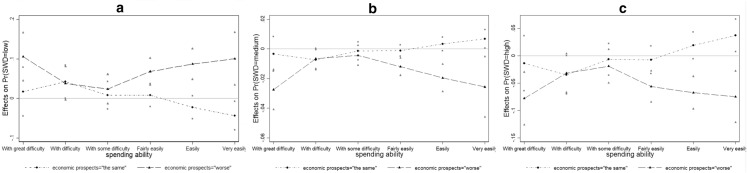
Marginal effect of expectations about future economic prospects at different levels of spending ability on SWD low (**a**). medium (**b**) and high (**c**). (“expectations about future economic prospects = better” base category)

To test H2, in model (5) we check how respondents' expectations about future economic prospects correlate with SWD at different levels of *spending_ability.* Such an analysis is carried out by using a specification where the two covariates interact. To correctly evaluate the impact of the two interacting terms, looking at the resulting marginal effects is essential (Brambor et al., 2006). Figure 2 shows how the *worse* and *the same* expectations about future economic prospects affect low (a), medium (b), and high (c) levels of SWD at different levels of spending ability. The effect of these expectations is calculated as compared with the *better* expectations about future economic prospects (which is the base category).

The results seem to suggest that having pessimistic economic prospects decreases (increases) the probability of being highly (poorly) satisfied with democracy especially among those who are placed at the extremes of the spending ability scale, i.e., among those who have great economic difficulty and those who make ends meet very easily. This finding is partially consistent with H2 that predicted an effect of bad economic prospects only among the poorest people.

## Discussion and conclusion

Our empirical analysis supports the idea that citizens’ expectations about future economic prospects are correlated with SWD (H1). According to our findings, and in line with previous literature (Loveless & Binelli, 2020; Nadeau et al., 2019), when citizens have pessimistic (optimistic) expectations about their future economic status, they are less (more) satisfied with democracy.

These findings are particularly relevant during the COVID-19 pandemic that has profoundly shaped citizens’ economic expectations (Fetzer et al., 2020). Indeed, our analyses suggest that any effect on expectations of future economic deterioration is likely to matter for SWD. In this perspective, in the aftermath of the outbreak, governing authorities should pay greater attention to the political attitudes of those individuals.

The effect of expectations on SWD does not seem to occur uniformly across the total citizenry. Instead, it appears to be particularly evident among those at the extremes of the spending ability distribution. This means that disappointment with democracy can be particularly hard among the marginalized people, who risk being trapped in their negative economic perceptions if no actions are taken to improve their expectations, and among rich people that believe to be severely hit by the pandemic. In line with Nadeau et al., (2019, p. 1083), we confirm that the strength of the effect of economic prospects on SWD “depends on one’s place on the socioeconomic ladder”. However, we further advance the literature by demonstrating that economic expectations play a significant role in explaining SWD, especially for more indigent and wealthier citizens. This result provides an interesting contribution to the literature exploring the political consequences of the COVID-19 crisis by evidencing a possible characterization of people’s reactions to disease-related economic threats.

Our analysis also adds further insights to the general discussion on possible procedural inputs and outputs conditioning SWD by shedding additional light on the main potential factors behind different individuals’ support across countries in times of shocks (Bol et al., 2020; Naumann et al., 2020; Vlachová, 2019). All in all, the study suggests that the capacity to ensure people’s satisfaction with the performance of democracy might depend on governments’ ability to intercept the needs of the population, which are not homogeneous but stratified based on their ability to withstand economic shocks.

## Footnotes

1 https://ecfr.eu/publication/together_in_trauma_europeans_and_the_world_after_covid_19/ [accessed on 15/9/2020]; https://www.eurofound.europa.eu/publications/report/2020/living-working-and-covid-19 [accessed on 6/11/2020].

2 Specifically “by publishing the link to the survey on social media and distributing it among Eurofound’s contacts and stakeholders, complemented by social media advertising, targeting hard-to-reach groups” (Sandor and Ahrendt, 2020, p. 1).

3 Where low includes values from 1 to 3, medium from 4 to 7 and high from 8 to 10. Results obtained when relying on the original variable are not qualitatively different from those reported in the following section. They are not included in the paper to save space but can be requested from the authors.

4 It is worth noting that differently from Nadeau et al. (2019)’s MWB index, our *spending_ability* variable does not distinguish between household income and assets.
